# Clinical significance and predictive factors of delayed gastric conduit emptying after esophagectomy

**DOI:** 10.1007/s00595-026-03371-4

**Published:** 2026-07-02

**Authors:** Yuta Sato, Yoshihiro Tanaka, Yuji Hatanaka, Seito Fujibayashi, Noriki Mitsui, Ryoma Yokoi, Takeshi Horaguchi, Keita Matsumoto, Masahiro Fukada, Itaru Yasufuku, Ryuichi Asai, Jesse Yu Tajima, Nobuhisa Matsuhashi

**Affiliations:** 1 Department of Gastroenterological Surgery and Pediatric Surgery, Gifu University Graduate School of Medicine, 1-1 Yanagido, Gifu City, 501-1194 Gifu Prefecture Japan; 2 Department of Clinical Anatomy Development Studies, Gifu University Graduate School of Medicine, 1-1 Yanagido, Gifu City, 501- 1194 Gifu Prefecture Japan

**Keywords:** Esophagectomy, Delayed gastric conduit emptying, Severity classification, Postoperative complications, Body weight loss

## Abstract

**Purpose:**

Delayed gastric conduit emptying (DGCE) is a functional complication after esophagectomy that can cause nutritional and respiratory issues, but its risk factors remain unclear.

**Methods:**

We retrospectively analyzed 166 patients after esophagectomy at Gifu University Hospital (2020–2024). DGCE was diagnosed per International Society for Diseases of the Esophagus criteria and graded using a novel radiographic classification (Grade 1–3). Clinical and postoperative outcomes were compared, and risk factors analyzed by logistic regression.

**Results:**

DGCE occurred in 29 patients (17.5%). Early DGCE patients were more often female (*p* = 0.005), had greater gastric conduit–mediastinum length difference (*p* < 0.001), and fewer fixation sutures (*p* < 0.001). No independent predictors were identified, though female sex and higher body mass index (BMI) showed trends. Pneumonia and late DGCE were more frequent (both *p* < 0.001). Severe DGCE (Grade 3) caused greater weight loss at 6 months and 1 year (*p* = 0.013, *p* < 0.001).

**Conclusions:**

Post-esophagectomy DGCE was linked to excessive conduit length and insufficient fixation. Careful intraoperative handling and structured long-term follow-up are crucial.

**Supplementary Information:**

The online version contains supplementary material available at https://doi.org/10.1007/s00595-026-03371-4.

## Introduction

Recent advances in minimally invasive surgery have improved the surgical outcomes of esophageal cancer, and postoperative functional complications have become an important clinical concern [1]. Delayed gastric conduit emptying (DGCE), which refers to the delayed passage of fluid and food through the reconstructed gastric conduit, can cause symptoms such as nausea, vomiting, early satiety, and reflux, potentially leading to poor oral intake and deterioration of quality of life (QOL). Reported incidences of DGCE vary widely, ranging from 2.2% to 47%, largely due to inconsistencies in definitions, diagnostic criteria, and the timing of assessment [2]. In 2020, the International Society for Diseases of the Esophagus (ISDE) developed an international expert consensus on DGCE using a modified Delphi process [3]. According to this consensus, the diagnosis of DGCE requires the presence of clinical symptoms (e.g., nausea, vomiting, early satiety, or bloating), imaging findings (e.g., contrast studies, endoscopy, or computed tomography [CT]) demonstrating gastric conduit stasis, and the exclusion of mechanical obstruction (e.g., anastomotic stricture). DGCE is classified according to the timing of onset into “early DGCE” (within 14 days after surgery) and “late DGCE” (after 14 days), and symptom severity is graded on a four-point scale from Grade I to IV. In 2025, Schneider et al. highlighted the clinical heterogeneity of DGCE and proposed a detailed classification based on its underlying causes and pathophysiology. In this system, DGCE is divided into four classes: Class I (functional DGCE), Class II (conduit-related DGCE), Class III (hiatal DGCE), and Class IV (other). The classification considers various causes, including impaired gastric conduit motility, anatomical abnormalities, mechanical issues at the diaphragmatic hiatus, and postoperative adhesions or tumor infiltration [4]. Although the standardization of diagnostic criteria for DGCE has been progressing, considerable variability and unresolved issues remain regarding its actual incidence, risk factors, and clinical impact. Thus, the clinical significance of radiographically assessed conduit distension as an indicator of DGCE severity remains unclear.

This study aimed to clarify the incidence, risk factors, and clinical consequences of DGCE after esophagectomy. We performed a retrospective cohort analysis using a novel three-tier severity classification based on imaging findings.

## Methods

### Study design and patients

This single-center retrospective cohort study included all consecutive patients who underwent esophagectomy at Gifu University Hospital between January 1, 2020, and December 31, 2024. The study population included cases reconstructed using our standard procedure: cervical hand-sewn anastomosis with a subtotal gastric conduit elevated via the posterior mediastinal route. The exclusion criteria were as follows: Ivor Lewis reconstruction, retrosternal route reconstruction, reconstructions other than the gastric conduit, mechanical anastomosis, and cases requiring postoperative reintubation or noninvasive positive pressure ventilation (respiratory support may affect gastric conduit motility and radiological assessment).

## Surgical procedure

**Fig. 1:**
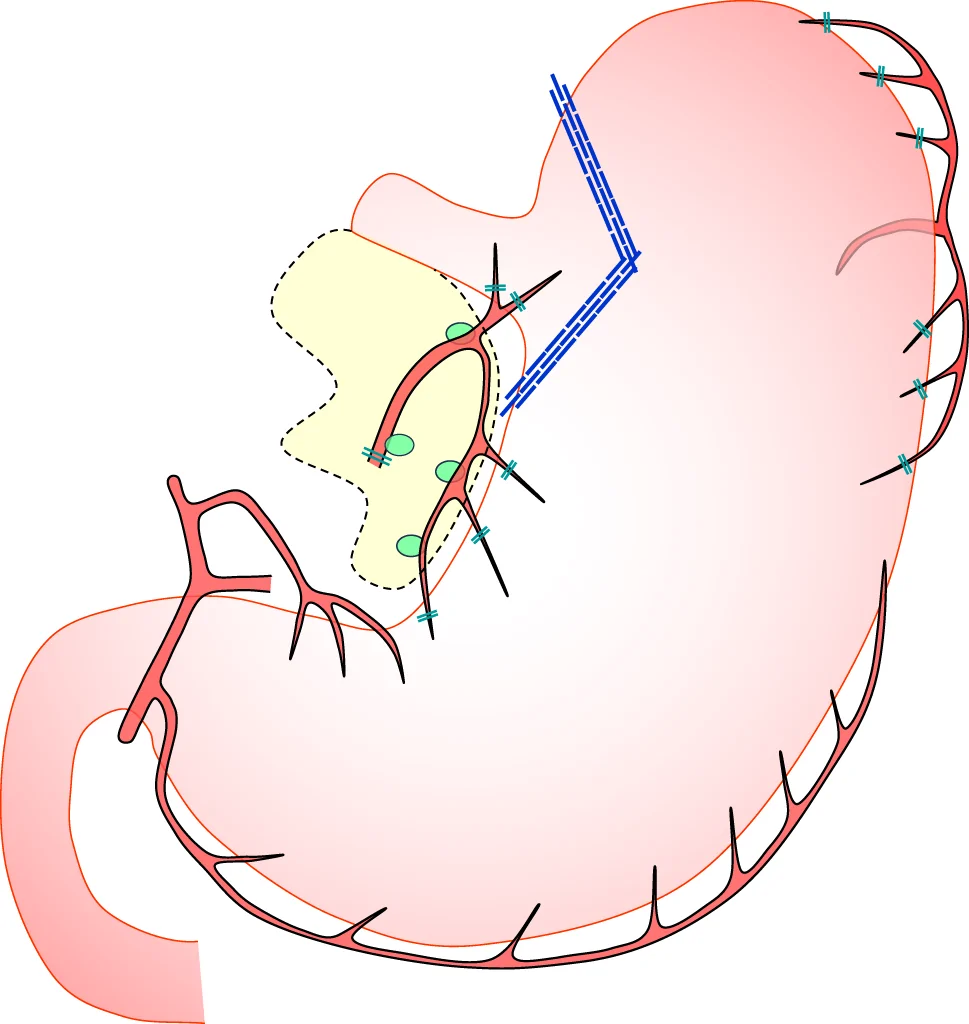
Standardized gastric conduit creation technique. The left gastric artery inflow territory was resected while preserving the right gastric artery territory. The stomach was transected using two 60-mm linear staplers (blue dashed lines)

**Fig. 2:**
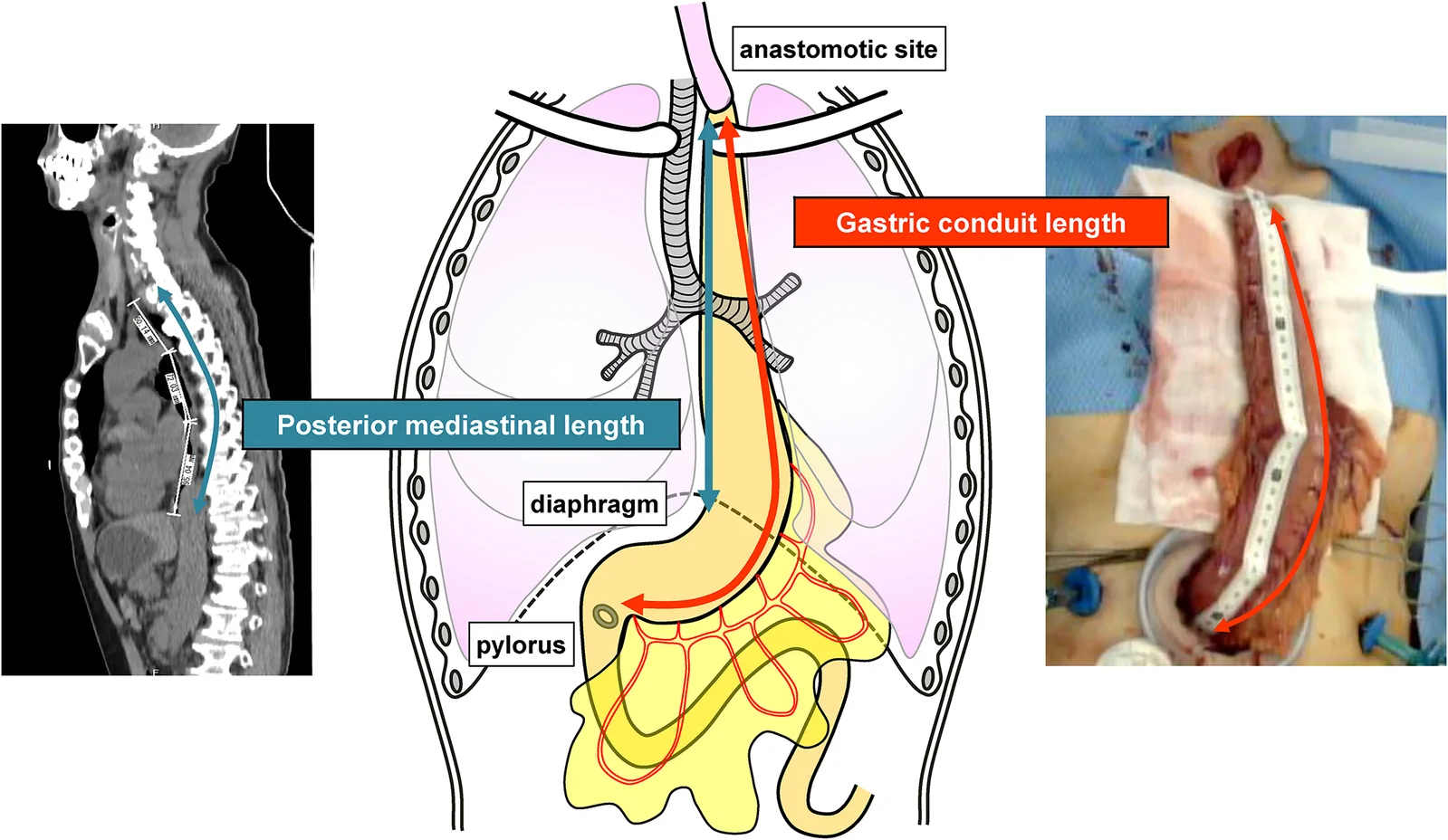
Measurement of the subtotal gastric conduit and the posterior mediastinal lengths

The gastric conduit was created using a standardized technique in all patients. The inflow territory of the left gastric artery was resected, while the right gastric artery territory was preserved. A minimum margin of 30 mm from the tumor was ensured, and the stomach was transected using two 60-mm linear staplers (Fig. 1). After hand-sewn anastomosis, the gastric conduit was consistently secured to the diaphragmatic crura along its ventral aspect with evenly spaced sutures approximately 1 cm apart (maximum of 7). The conduit length was measured intraoperatively along the greater curvature from the planned anastomotic site to the pylorus. The posterior mediastinal length was measured on preoperative sagittal CT images or intraoperatively from the supraclavicular region to the diaphragmatic crura (Fig. 2). All patients received postoperative mechanical ventilation via endotracheal intubation on the day of surgery and were extubated the following morning, followed by oxygen therapy via an inspiratory mask. In all cases, a nasogastric tube was placed after anastomosis and routinely removed on the day after surgery. In addition, a jejunostomy was created in all patients at 30 cm distal to the ligament of Treitz, and postoperative enteral nutrition was provided using a standardized regimen. To prevent postoperative bowel obstruction, all jejunostomies were removed by three months after surgery at the latest. Postoperative analgesia and fluid management were performed according to standardized protocols based on patient weight and hemodynamics, and no prokinetic agents were administered to any patient.

## DGCE diagnosis

DGCE was diagnosed according to the ISDE criteria, based on clinical symptoms such as oral intake intolerance, early satiety, bloating, nausea/vomiting, or reflux, after excluding mechanical obstruction (including anastomotic stricture, pyloric obstruction, or conduit route abnormality) [3]. Early DGCE was defined as occurring within 14 days after surgery, and late DGCE as occurring between 14 days and one year after surgery. In cases of early DGCE, nasogastric tube reinsertion was performed. No endoscopic procedures or additional surgeries (e.g., pyloric dilation) were performed in any patient.

## Radiographic grading of early delayed gastric conduit emptying

**Fig. 3:**
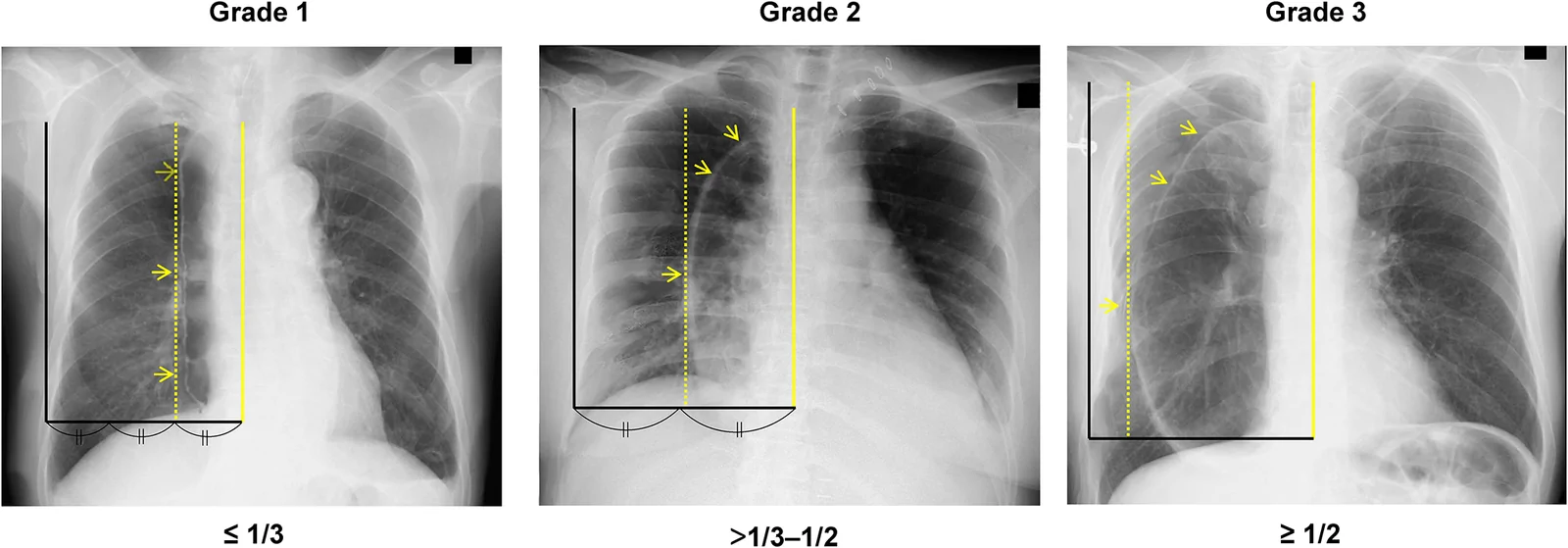
Early DGCE graded by maximal intrathoracic gastric conduit expansion, according to our classification

For early DGCE cases, chest radiographs taken within 14 days postoperatively were used to measure the maximal intrathoracic expansion (width) of the gastric conduit. Grading was defined as follows: Grade 1, ≤ 1/3 of the right (or left) thoracic cavity; Grade 2, > 1/3 to 1/2; Grade 3, ≥ 1/2 (Fig. 3). Chest radiographs were obtained for all patients in the standing position at a fixed time in the morning. The radiographic assessment was independently performed by two investigators who were blinded to the patients’ clinical information and outcomes, and no significant discrepancies were observed between their measurements.

## Outcome measures

The primary outcomes were the incidence of DGCE and the identification of associated risk factors. Secondary outcomes included clinical and operative factors associated with DGCE, stratified by severity.

### Statistical analysis

All analyses were performed using R (ver. 4.5.1). Continuous variables were expressed as the mean ± standard deviation (SD) for normally distributed data, or as the median (interquartile range) for non-normally distributed data. Categorical variables were presented as counts and percentages. Comparisons between the DGCE and non-DGCE groups were performed using Student’s *t*-test or the Mann–Whitney U test for continuous variables, and the chi-square or Fisher’s exact test for categorical variables. To identify factors associated with DGCE, univariable logistic regression analyses were performed first, followed by a multivariable analysis including the following clinically relevant variables: sex, tumor location, pathological T/N/Stage, neoadjuvant chemotherapy, mediastinal length, conduit length, conduit–mediastinal length difference, number of fixation sutures, and body mass index (BMI). Due to the small number of DGCE events, Firth’s penalized logistic regression was applied to reduce small-sample bias. Two-sided p-values of < 0.05 were considered statistically significant. ROC curve analysis was performed to determine the optimal cutoff value of the gastric conduit–mediastinum length difference for predicting early DGCE.

## Results

**Fig. 4:**
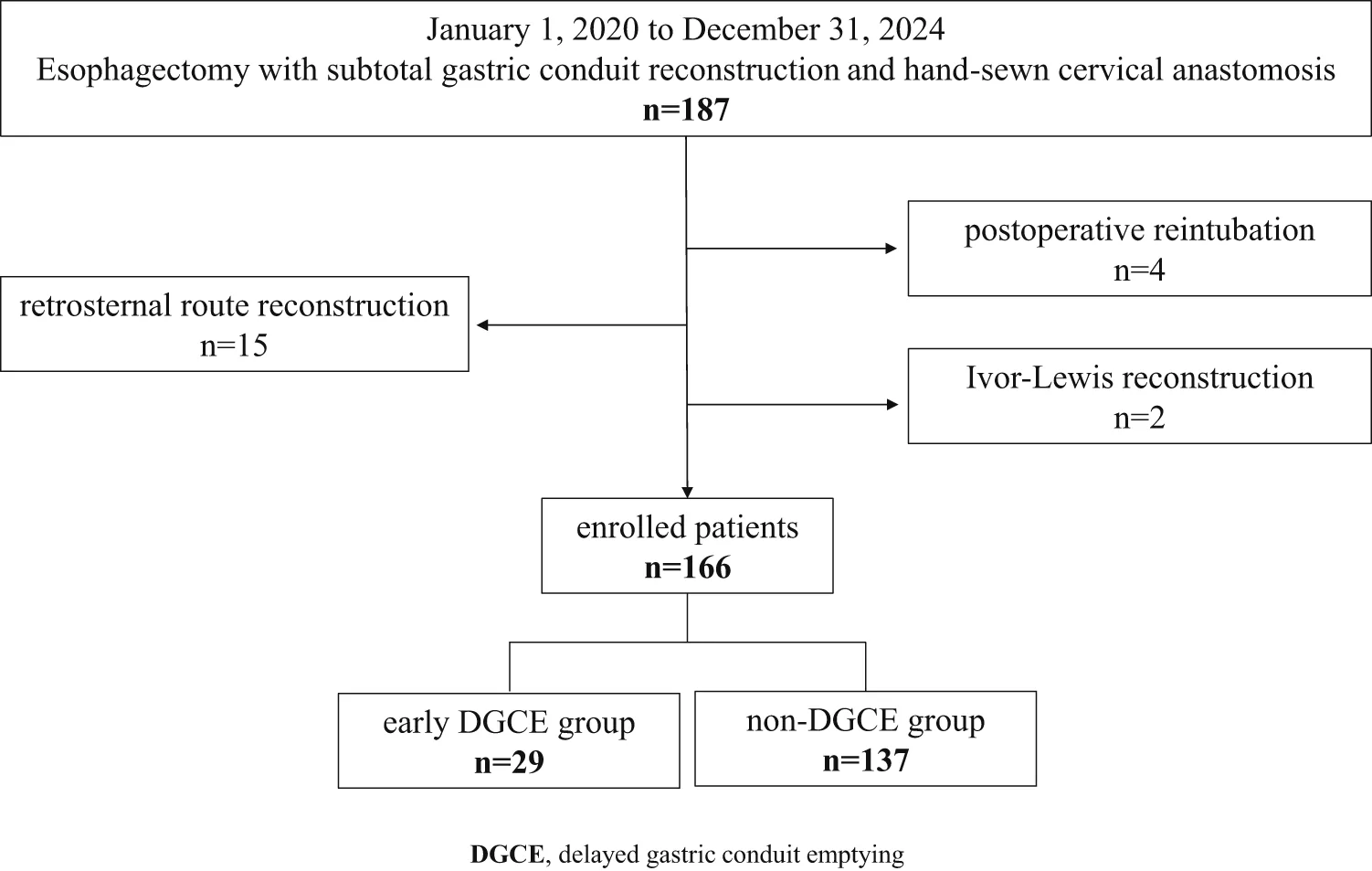
CONSORT diagram of patient selection

During the study period, a total of 187 esophagectomies were performed, of which 166 cases underwent reconstruction using our standard approach (subtotal gastric conduit with cervical hand-sewn anastomosis) and were included in the analysis, with a mean follow-up period of 21 ± 3 months. (Fig. 4). Patient characteristics are summarized in Table 1. The median age of all patients was 70 years (range, 63–76 years), with 132 male patients (79.5%) and 34 female patients (20.5%). The median BMI was 21.8 kg/m² (range, 19.6–23.4 kg/m²). The tumor location was the cervical to upper thoracic esophagus in 35 cases (21.1%), the middle thoracic esophagus in 77 cases (46.4%), and the lower thoracic to abdominal esophagus in 54 cases (32.5%). Neoadjuvant chemotherapy was administered in 113 patients (68.1%). Histologically, 140 cases (84.3%) were classified as squamous cell carcinoma, and 26 (15.7%) as adenocarcinoma. The median subtotal gastric conduit length was 34 cm (range, 32–35 cm), the posterior mediastinal length was 25.5 cm (range, 23–28 cm), the median difference between gastric conduit length and mediastinal length was 8.5 cm (range, 7.1–10.8 cm), and the median number of sutures used to fix the gastric conduit to the diaphragmatic crura was 5 (range, 4–7). The median body weight reduction was 7.46% (range, 3.0–10.0%) at 6 months postoperatively and 8.59% (range, 5.0–15.2%) at 1 year postoperatively. Anastomotic leakage occurred in 3 patients (1.8%), anastomotic stenosis in 16 patients (9.6%), and postoperative pneumonia in 23 patients (13.9%). Early and late DGTE were observed in 29 cases and 13 cases, respectively. Notably, the mean posterior mediastinal length was significantly longer in male patients than in female patients (male vs. female: 25.35 ± 2.18 cm vs. 24.00 ± 2.46 cm; *t*-test, *p* = 0.0055, Supplementary Table 1).

**Table 1:** Baseline patient characteristics and perioperative data

Characteristics	Data (*n*=166)
Age (median [IQR]), years	70 [63-76]
Sex (Male/Female), n (%)	132 (79.5) / 34 (20.5)
Body height (median [IQR]), cm	165.9 [159.9–170.6]
Preoperative weight (median [IQR]), kg	58.7 [51.0–64.2]
Body mass index (median [IQR]), kg/m^2^	21.8 [19.6–23.4]
Tumor location (Ce, Ut / Mt / Lt, Ae)	35 / 77 / 54
Clinical T (UICC ver.8)	
1a	18
1b	52
2	25
3	38
4a	26
4b	7
Clinical N (UICC ver.8)	
0	75
1	58
2	24
3	9
Clinical M (UICC ver.8)	
0	158
1	8
Neoadjuvant chemotherapy, n (%)	113 (68.1)
Pathology (SCC / Adenocarcinoma), n (%)	140 (84.3) / 26 (15.7)
Gastric conduit length (median [IQR]), cm	34 [32–35]
Posterior mediastinal length (median [IQR]), cm	25.5 [23–28]
Gastric conduit – Mediastinum difference, (median [IQR]) cm	8.5 [7.1–10.8]
Number of sutures (median [IQR])	5 [4–7]
Anastomotic leakage, n (%)	3 (1.8)
Anastomotic stenosis, n (%)	16 (9.6)
Pneumonia, n (%)	23 (13.9)
Early DGCE, n (%)	29 (17.5)
Late DGCE, n (%)	13 (7.8)
Length of hospital stay (median [IQR]), days	24 [18–32]
Weight loss at 6 months (median [IQR]), %	7.46 [3.0–10.0]
Weight loss at 1 year (median [IQR]), %	8.59 [5.0–15.2]

Values are presented as median [interquartile range, IQR] as appropriate

Ce, cervical; Ut, upper thoracic; Mt, middle thoracic; Lt, lower thoracic; Ae, abdominal esophagus; SCC, squamous cell carcinoma; DGCE delayed gastric conduit emptying

Patient demographics and perioperative data for the early DGCE and non-DGCE groups are summarized in Table 2. There was no significant difference in age (mean ± SD) between the two groups (data shown as early DGCE vs. non-DGCE): (67.7 ± 10.2 vs. 69.6 ± 10.2 years; *p* = 0.351). The early DGCE group had a higher proportion of females in comparison to the non-DGCE group (female: 41.4% vs. 16.1%; *p* = 0.005). No significant differences were observed between the groups with respect to tumor location, clinical stage, histological type, or administration of neoadjuvant chemotherapy. Regarding surgical and perioperative factors, the early DGCE group had a significantly shorter posterior mediastinal length (23.3 ± 2.4 cm vs. 25.5 ± 2.1 cm; *p* < 0.001), a greater difference between gastric conduit length and mediastinal length (10.3 ± 1.6 cm vs. 7.9 ± 2.2 cm; *p* < 0.001), and fewer sutures used to fix the gastric conduit to the diaphragmatic crura (4.0 ± 1.3 vs. 5.3 ± 1.1; *p* < 0.001). No significant differences were observed in patient height, gastric conduit length, postoperative hospital stay, BMI, or postoperative body weight reduction at the timepoints of 6 months and 1 year postoperatively. Regarding postoperative complications, the incidence rates of pneumonia (37.9% vs. 8.8%; *p* < 0.001) were higher in the early DGCE group, whereas the rates of anastomotic leakage and stenosis did not differ significantly between the groups.

**Table 2:** Patient characteristics and perioperative data by DGTE status

	non-DGCE group	early DGCE group	*p*-Value
	*n*=137	*n*=29	
Age (mean ± SD), years	69.6 ± 10.2	67.7 ± 10.2	0.351
Male, n (%)	115 (83.9)	17 (58.6)	0.005*
Female, n (%)	22 (16.1)	12 (41.4)	
Body height (mean ± SD), kg/m^2^	164.3 ± 8.9	167.7 ± 8.0	0.061
Preoperative weight (mean ± SD), kg	63.6 ± 61.9	62.3 ± 9.8	0.910
Body mass index (mean ± SD), kg/m^2^	23.6 ± 2.5	22.0 ± 2.3	0.732
Tumor location (Ce, Ut / Mt / Lt, Ae)	27/63/47	8/14/7	0.474
Clinical stage (1/2/3/4a/4b) (UICC ver.8)	50/24/26/32/5	9/2/11/6/1	0.210
Neoadjuvant chemotherapy, n (%)	91 (66.4)	22 (75.9)	0.441
Pathology (SCC / Adenocarcinoma)	122/17	25/4	1.000
Gastric conduit length (mean ± SD), cm	33.3 ± 2.0	33.6 ± 1.5	0.489
Posterior mediastinal length (mean ± SD), cm	25.5 ± 2.1	23.3 ± 2.4	<0.001*
Gastric conduit – Mediastinum difference (mean ± SD), cm	7.9 ± 2.2	10.3 ± 1.6	<0.001*
Gastric conduit width (mean ± SD), cm	6.2 ± 0.8	6.3 ± 1.1	0.887
Number of sutures (mean ± SD)	5.3 ± 1.1	4.0 ± 1.3	<0.001*
Anastomotic leakage, n (%)	1 (0.7)	2 (6.9)	0.134
Anastomotic stenosis, n (%)	13 (9.5)	3 (10.3)	1.000
Pneumonia, n (%)	12 (8.8)	11 (37.9)	<0.001*
Late DGCE, n (%)	0 (0)	13 (44.8)	<0.001*
Length of hospital stay (mean ± SD), days	28.54 ± 13.98	28.59 ± 13.86	0.987
Weight loss at 6 months (mean ± SD), %	8.30 ± 8.90	9.23 ± 6.05	0.594
Weight loss at 1 year (mean ± SD), %	9.16 ± 8.42	11.35 ± 4.92	0.180

Values are presented as mean ± standard deviation (SD)

Postoperative complications are classified according to Clavien-Dindo grade ≥ II

Ce, cervical; Ut, upper thoracic; Mt, middle thoracic; Lt, lower thoracic; Ae, abdominal esophagus; SCC, squamous cell carcinoma; DGCE delayed gastric conduit emptying

**Fig. 5:**
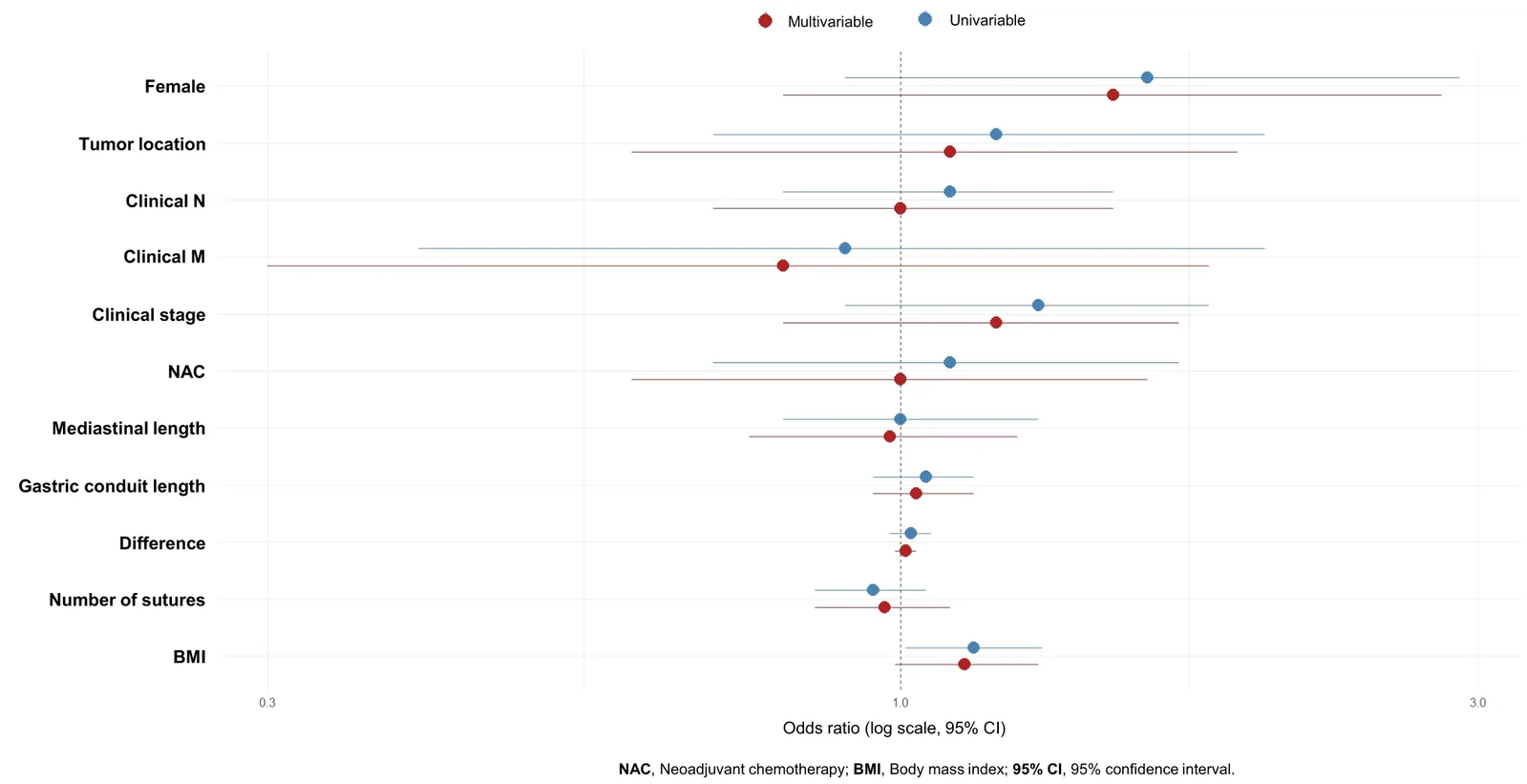
Forest plot of multivariable Firth-corrected logistic regression for early DGCE

To identify factors associated with the development of the early DGCE, univariable analyses were first conducted. The results suggested a trend toward an increased risk of early DGCE in patients with higher BMI (odds ratio [OR], 1.02; 95% CI, 0.99–1.04; *p* = 0.08) (Table 3). Subsequently, a multivariable Firth-corrected logistic regression analysis including sex, tumor location, N factor, M factor, clinical stage, neoadjuvant chemotherapy, mediastinal length, gastric conduit length, the difference between gastric conduit and mediastinum, number of sutures between the gastric conduit and diaphragmatic crura, and BMI was performed. No independent predictive factors reached statistical significance; however, female sex (adjusted OR, 2.08; 95% CI, 0.10–43.0) showed a trend toward an association with an increased risk of DGCE (Fig. 5).

**Table 3:** Factors associated with early DGCE: Univariable and Multivariable Firth Logistic Regression

	Univariable OR (95% CI)	*p*-Value	Multivariable OR (95% CI)	*p*-Value
Sex (male = reference)	3.84 (0.17–86.5)	0.52	2.08 (0.10–43.0)	0.73
Body mass index (per kg/m^2)^	1.02 (0.99–1.04)	0.08	1.01 (0.99–1.04)	0.16
Posterior mediastinal length (per cm)	0.69 (0.16–3.0)	0.57	0.72 (0.18–2.9)	0.68
Gastric conduit length (per cm)	0.94 (0.25–3.6)	0.93	0.90 (0.24–3.4)	0.88
Gastric conduit – Mediastinum difference (per cm)	0.76 (0.25–2.3)	0.67	0.72 (0.24–2.2)	0.62
Number of sutures	0.90 (0.17–4.9)	0.93	0.84 (0.16–4.3)	0.88

OR: odds ratio; 95% CI: 95% confidence interval

OR represents the odds of DGCE relative to the reference for categorical variables or per unit increase for continuous variables (BMI per 1 kg/m², mediastinal and conduit lengths per 1 cm, conduit–mediastinum difference per 1 cm). Male is the reference for sex. Firth’s logistic regression was used to reduce bias from limited events. *p* < 0.05 considered significant

**Fig. 6:**
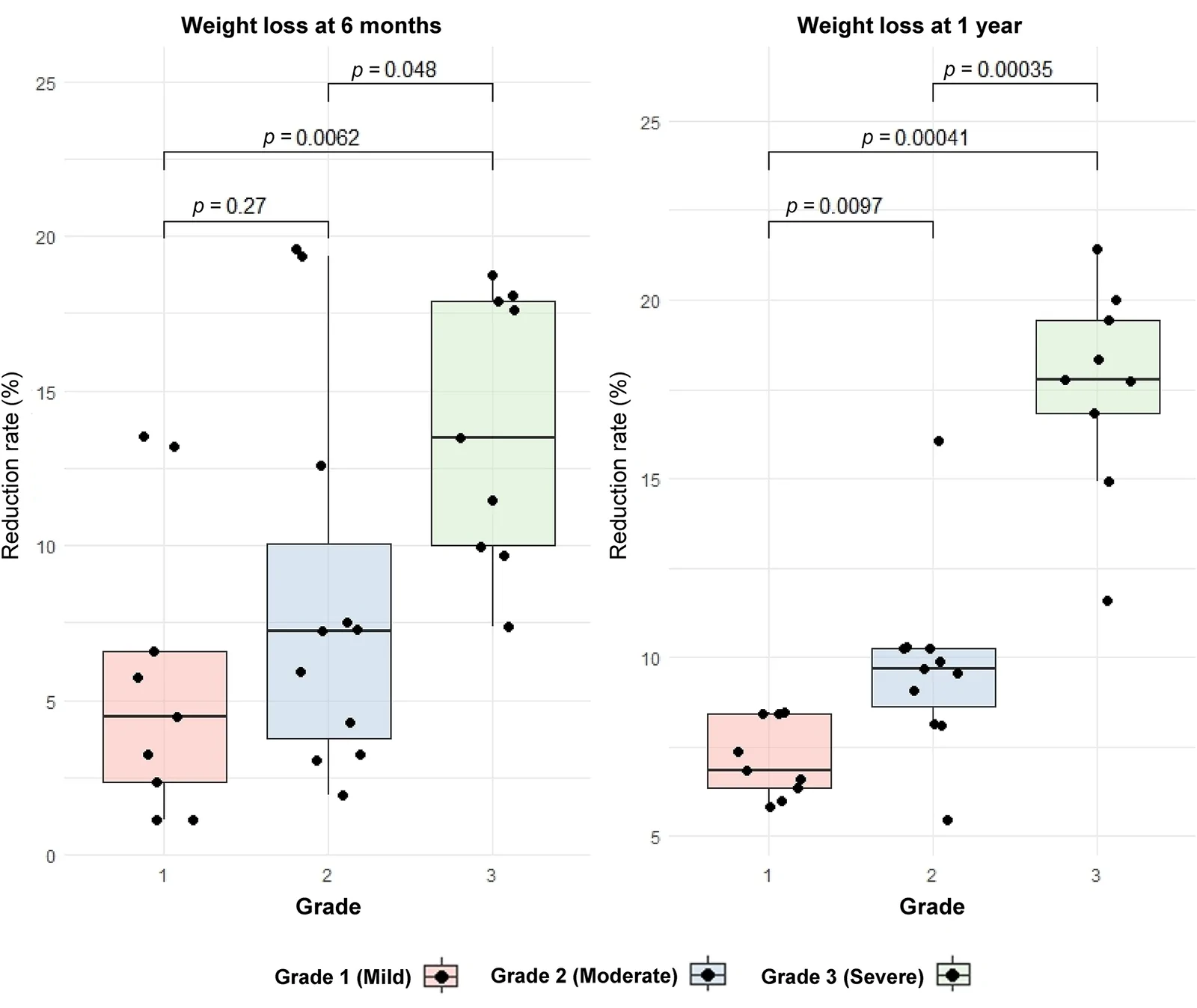
Weight loss (%) by early DGCE grade at 6 months and 1 year postoperatively

The early DGCE cohort (*n* = 29) was stratified by severity grade (Fig. 2), and subgroup analyses were conducted among patients with Grade 1 (mild), Grade 2 (moderate), and Grade 3 (severe) DGCE (Table 4). The mean (± SD) preoperative BMI did not differ significantly among the grades (Grade 1: 21.7 ± 3.8 kg/m²; Grade 2: 22.3 ± 2.2 kg/m²; Grade 3: 22.1 ± 2.1 kg/m²; Kruskal–Wallis test, *p* = 0.903). Six months postoperatively, the rate of weight loss was significantly greater in Grade 3 in comparison to Grade 1 (Wilcoxon rank-sum test, Bonferroni-adjusted *p* = 0.0062). At one year postoperatively, weight loss differed significantly across all grades (Grade 1 vs. Grade 2: *p* = 0.0097; Grade 1 vs. Grade 3: *p* = 0.00041; Grade 2 vs. Grade 3: *p* = 0.00035) (Fig. 6). The analysis of postoperative complications revealed that pneumonia occurred in all Grade 3 patients, in 18% of Grade 2 patients, and in none of the Grade 1 patients (Fisher’s exact test, *p* < 0.001). The incidence of late DGCE was also significantly higher in Grade 3 (*p* = 0.003), whereas the rates of anastomotic leakage and stenosis did not differ significantly among the grades.

**Table 4:** Postoperative weight change and complications according to DGCE severity

	Grade 1 (Mild) *n*=9	Grade 2 (Moderate) *n*=11	Grade 3 (Severe) *n*=9	*p*-Value
Body mass index (mean ± SD), kg/m^2^	21.7± 3.8	22.3± 2.2	22.1± 2.1	0.904
Anastomotic leakage	0%	9%	11%	1.00
Anastomotic stenosis	0%	27%	0%	0.091
Pneumonia	0%	18%	100%	<0.001*
Late DGCE	11%	36%	89%	0.003*
Weight loss at 6 months (mean ± SD), %	5.7 ± 4.7	8.4 ± 6.2	13.8 ± 4.4	0.013*
Weight loss at 1 year (mean ± SD), %	7.1 ± 1.1	9.7 ± 2.5	17.6 ± 2.9	<0.001*

DGCE, delayed gastric conduit emptying

Statistical significance was assessed using Kruskal–Wallis test, Wilcoxon rank-sum test with Bonferroni adjustment for multiple comparisons (*), or Fisher’s exact test, as appropriate

## Discussion

DGE is a well-recognized postoperative complication following pancreaticoduodenectomy and gastrectomy and has been reported to prolong hospital stay [5, 6], impair postoperative QOL [7], and negatively affect long-term survival [8]. DGCE can also occur with notable frequency after esophagectomy, in which gastric conduit reconstruction is the standard procedure [9–11]. However, there is no clear consensus regarding optimal management strategies, including endoscopic interventions such as balloon dilation or botulinum toxin injection [12–14] and preventive surgical techniques aimed at facilitating gastric drainage [15–18]. Several studies have suggested that DGCE is associated with various complications, including pneumonia [19, 20], and may adversely affect hospital stay [21, 22] and postoperative QOL [20].

With reductions in anastomotic leakage rates achieved through refinements in surgical technique [23], the overall safety of esophagectomy has gradually improved. In this context, our study was conducted to further improve postoperative outcomes by focusing on the functional aspects of gastric conduit reconstruction. In the present study, patients with the early DGCE were more likely to be female and demonstrated a greater discrepancy between the lengths of the gastric conduit and mediastinum, as well as fewer fixation sutures between the conduit and the diaphragmatic crus. ROC curve analysis indicated that a gastric conduit–mediastinum length difference exceeding 9.05 cm was associated with an increased risk of early DGCE (sensitivity 93%, specificity 62%, area under the curve [AUC] = 0.809; see Supplementary Fig. 1). These findings suggest that an imbalance between conduit length and mediastinal distance, combined with insufficient fixation of the gastric conduit, may contribute to the development of DGCE. Previous reports have shown that females have greater gastric distensibility and slower gastric emptying than males [24, 25], suggesting that anatomical and physiological differences, rather than sex alone, contribute to the higher incidence of DGCE in female patients. While such distensibility is advantageous for creating a longer gastric conduit, excessive elongation may conversely increase the risk of delayed emptying. In the early DGCE group, the incidence of postoperative pneumonia was significantly higher, suggesting that gastric content stasis and an increased risk of aspiration due to impaired emptying may underlie the development of postoperative respiratory complications.

In the severe DGCE group (Grade 3 of our classification), the rates of body weight loss at 6 months and 1 year postoperatively were significantly greater than those in the other groups, suggesting deterioration of the postoperative nutritional status and delayed overall recovery [26–30]. DGCE is thought to result from a combination of anatomical abnormalities, vagal denervation, and impaired gastric motility [3–5], which may contribute to its persistence and progression. In our study, a substantial proportion of patients who developed severe early DGCE subsequently progressed to late DGCE. One possible explanation is that a dilated and angulated gastric conduit within the thoracic cavity may become adherent to the posterior mediastinal tissues or lung parenchyma, thereby leading to prolonged dysfunction. These findings underscore the importance of careful long-term follow-up in patients with severe early DGCE. Although differences in postoperative weight loss were observed according to the severity of DGCE, all patients received standardized short-term enteral nutrition. Therefore, the potential impact of long-term nutritional interventions on weight maintenance could not be assessed. The multivariable analysis in our study failed to identify any independent predictive factors, likely due to the small number of DGCE events and the complex interrelationships among the variables, indicating that multiple interacting factors may contribute to the onset of DGCE. Additional analyses confirmed that no significant multicollinearity was present. Nonetheless, the observation that DGCE was more frequently observed in female patients and in those with higher BMI is noteworthy and warrants validation in larger prospective studies.

This study had several limitations. First, it was a single-center, retrospective study, and potential selection and information biases could not be eliminated. Second, unmeasured confounding factors, such as variations in anesthetic management, postoperative analgesic use, and detailed assessments of the swallowing function, may have influenced the outcomes.

## Conclusion

In this study, DGCE was observed more frequently in female patients, especially when the gastric conduit was relatively long or insufficiently fixed. Severe DGCE tended to persist postoperatively and was associated with notable weight loss and pulmonary complications. These findings highlight the importance of careful intraoperative conduit handling. For female patients, those with a high BMI, and those with long gastric conduits, particular attention should be paid to intraoperative conduit fixation and avoiding excessive lengthening, which may be a simple yet effective measure to reduce the incidence of DGCE. Our proposed radiographic grading provides an objective framework for assessing the severity of early DGCE and may facilitate standardized evaluation across institutions.

## Supplementary Information

Below is the link to the electronic supplementary material.

Supplementary file

Supplementary file

## Data Availability

The datasets generated and analyzed during the current study are available from the corresponding author upon reasonable request.
