# Supplementary material for: Clinical significance and predictive factors of delayed gastric conduit emptying after esophagectomy
Source: Surg Today. 2026 Jul 2;56(10):2468–77. doi: 10.1007/s00595-026-03371-4 (PMC13558326; doi:10.1007/s00595-026-03371-4)
Supplement: Supplementary file 1 [file 595_2026_3371_MOESM2_ESM.pdf]

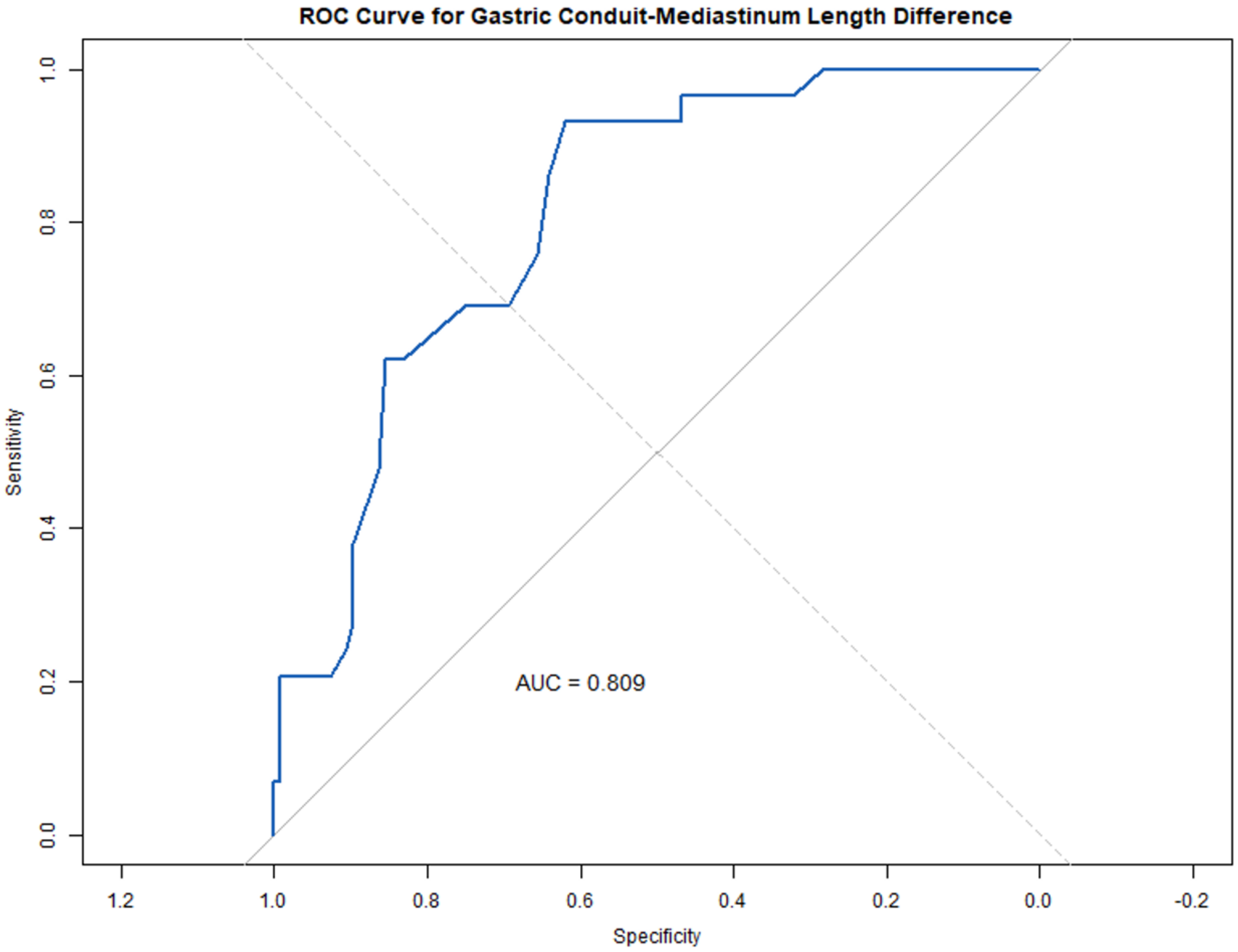
ROC Curve for Gastric Conduit-Mediastinum Length Difference
Sensitivity
Specificity
1.0
0.8
0.6
0.4
0.2
0.0
1.2
1.0
0.8
0.6
0.4
0.2
0.0
-0.2
AUC = 0.809
