# Supplementary material for: Clinical significance and predictive factors of delayed gastric conduit emptying after esophagectomy
Source: Surg Today. 2026 Jul 2;56(10):2468–77. doi: 10.1007/s00595-026-03371-4 (PMC13558326; doi:10.1007/s00595-026-03371-4)
Supplement: Supplementary file 2 [file 595_2026_3371_MOESM1_ESM.docx]

|  | n | mean ± SD (cm) | *p*-Value |
| --- | --- | --- | --- |
| Male | 132 | 25.35 ± 2.18 | 0.0055* |
| Female | 34 | 24.00 ± 2.46 |  |

**Supplementary Table 1.** Comparison of mediastinal length by sex.

SD, standard deviation. Statistical significance was assessed using Student’s t-test.

Comparison of mediastinal length between male and female patients in the present study cohort.
